# Fault Diagnosis of Static Eccentricity in Marine Diesel Generators Using 2D Short-Time Fourier Transform of Three-Phase Currents

**DOI:** 10.3390/s25247604

**Published:** 2025-12-15

**Authors:** Beom-Jin Joe, Jin-Sung Lee, Sang-Jae Yeo, Yong Jae Cho, Jee-Yeon Jeon

**Affiliations:** HD Korea Shipbuilding and Offshore Engineering, Seongnam-si 13553, Republic of Korea; beomjin.joe@hd.com (B.-J.J.); jinsung.lee@hd.com (J.-S.L.); yeo.sangjae@hd.com (S.-J.Y.); yongjae.cho@hd.com (Y.J.C.)

**Keywords:** marine diesel generator, two-dimensional short-time Fourier transform, current signal analysis, static eccentricity fault

## Abstract

Static eccentricity is an important early-stage fault in marine diesel generators, as small air-gap deviations caused by misalignment or mechanical wear can escalate into bearing damage and rotor–stator contact. To address the challenge of detecting such subtle faults, this study proposes a current signal analysis method based on the two-dimensional short-time Fourier transform (2D STFT) for early detection of static eccentricity faults in marine diesel generators. Using three-phase currents measured during normal operation and fault data synthesized with a physics-based electromechanical coupling model (1–5% eccentricity), we construct a two-dimensional phase–time representation rather than treating each phase as an independent one-dimensional time series and then apply 2D STFT. This formulation enables the simultaneous capture of inter-phase relationships and spatial patterns in the time–frequency–phase domain. Experiments indicate a distinct energy rise near 1020 Hz as static eccentricity increases. This trend enables the proposed method to distinguish small faults of approximately 5% eccentricity, which remain difficult to detect using conventional 1D STFT. As a result, the approach improves the diagnostic accuracy of non-contact, current-based monitoring for static eccentricity faults. Future work will include validation using real in-service fault data and extensions to other fault modes such as dynamic eccentricity and bearing defects.

## 1. Introduction

Marine diesel generators serve as the primary onboard power plants, and their importance has been increasing with the gradual transition of marine propulsion systems toward electric or hybrid electric architectures. Faults in these generators can lead directly to loss of thrust, operational delays, and increased maintenance costs, with substantial economic consequences and heightened safety risks. In particular, static eccentricity—arising from an asymmetric air gap between the rotor and stator—affects both the generator’s electromagnetic behavior and its mechanical vibration response, potentially resulting in catastrophic outcomes [1]. Accordingly, the development of technologies that enable the early detection and mitigation of static eccentricity in diesel generators is essential to ensure the safety and reliability of ship operations.

Generally, a variety of approaches have been proposed for generator fault diagnosis using signals such as vibration, acoustic emissions, air gap flux, and electrical quantities (current and voltage). Among these, current-based methods are widely adopted in industrial practice because they allow for stable data acquisition without interfering with equipment operation, thanks to their simple, non-intrusive sensor installation [2,3]. Over time, current signal-based diagnostics have evolved from time domain statistical analysis and frequency domain decomposition to time–frequency transforms and more recently to feature extraction techniques leveraging machine learning and deep learning [4,5]. However, many existing studies analyze single-phase currents independently or apply time–frequency transforms in a strictly one-dimensional manner, thereby failing to fully capture inter-phase relationships and spatial patterns inherent in three-phase systems [2].

Over the past decade, numerous studies have focused on diagnosing generator faults using one-dimensional Short-Time Fourier Transform (1D STFT) or Motor Current Signature Analysis (MCSA) methods. Oviedo et al. [6] demonstrated that conventional MCSA and 1D STFT approaches suffer from degraded sensitivity under variable load and speed conditions, as the spectral components of a single-phase current become masked by supply harmonics and negative-sequence currents. Similarly, Karami et al. [7] analyzed rotor asymmetry faults in a three-phase line-start permanent magnet synchronous motor and showed that single-phase current analysis fails to reflect inter-phase coupling effects, resulting in weak fault discrimination at early eccentricity stages. These findings highlight that relying solely on one-dimensional current analysis is inadequate for detecting low-level eccentricity or asymmetry faults, especially in marine diesel generators where low signal-to-noise ratios and overlapping harmonic components further obscure fault-related spectral features.

More recent investigations have attempted to improve diagnostic accuracy by applying hybrid time–frequency and deep-learning-based frameworks to current-signal analysis. Zhang et al. [8] employed a CNN–LSTM architecture to detect electrical machine faults using time–frequency representations, while Li et al. [9] utilized wavelet packet decomposition combined with convolutional neural networks for feature extraction and fault classification. Although such methods achieved improved classification performance, they still rely on one-dimensional or single-phase data inputs and thus cannot represent the inter-phase coupling phenomena inherent in three-phase systems. Consequently, existing approaches remain limited in their ability to capture spatial–temporal relationships between phase currents—an essential factor for detecting weak eccentricity or mixed electromechanical faults in marine applications.

Most current-based diagnostic methods either analyze a single phase or treat multi-phase signals independently so that only the temporal spectral content of each phase is utilized. As a result, spatial asymmetries and inter-phase coupling effects induced by static eccentricity are only indirectly reflected in the measured signals and may remain hidden within the dominant supply harmonics. In this study, we interpret the three-phase currents as a two-dimensional phase–time signal and apply a two-dimensional short-time Fourier transform (2D STFT). This formulation explicitly introduces a phase axis into the time–frequency representation, allowing for the extraction of phase-direction spectral components associated with air-gap asymmetry. By doing so, we aim to improve the sensitivity of current-based diagnostics to low-level static eccentricity in marine diesel generators.

In this study, normal operating current data collected from an actual marine diesel generator were used as the baseline, and static eccentricity fault data were generated through a physics-based electromechanical coupling model for analysis. Unlike previous approaches, the proposed method does not treat each phase current as an independent one-dimensional time series; instead, it constructs a two-dimensional phase–time signal from the three-phase currents and applies 2D STFT. This enables the capture of specific frequency components and their inter-phase relationships within the time–frequency–phase domain. The analysis revealed frequency components that were not apparent in conventional single-phase STFT, showing significant variation with the degree of static eccentricity, and demonstrated that even minor eccentricity levels of approximately 5% can be effectively detected. Considering that prior studies typically achieved detection only at eccentricity levels of 15–40% or higher, these findings confirm that the proposed method is well suited for early detection of static eccentricity [10,11].

The specific contributions of this work are summarized as follows.

(1) We construct static-eccentricity fault data for a marine diesel generator by combining long-term three-phase current measurements with a physics-based electro-mechanical model, thereby generating realistic low-level fault scenarios.
(2) We propose a 2D STFT formulation in which the three-phase currents are arranged into a phase–time matrix and analyzed simultaneously along the time and phase axes. This provides phase-axis spectral components that directly encode the inter-phase coupling and spatial asymmetry of the air-gap field, which are not available in conventional multi-phase MCSA or 1D STFT approaches.
(3) We show that, for the target generator, the 2D STFT yields a distinct harmonic component at 1020 Hz (17th harmonic of the 60 Hz supply), whose magnitude increases monotonically from 0% to 5% static eccentricity. In contrast, conventional 1D STFT analysis of each phase exhibits negligible variation under the same conditions.

These results indicate that the proposed method can enhance the sensitivity of current-based diagnostics to early-stage static eccentricity faults.

## 2. System Modeling for Eccentricity Fault Analysis

**Figure 1:**
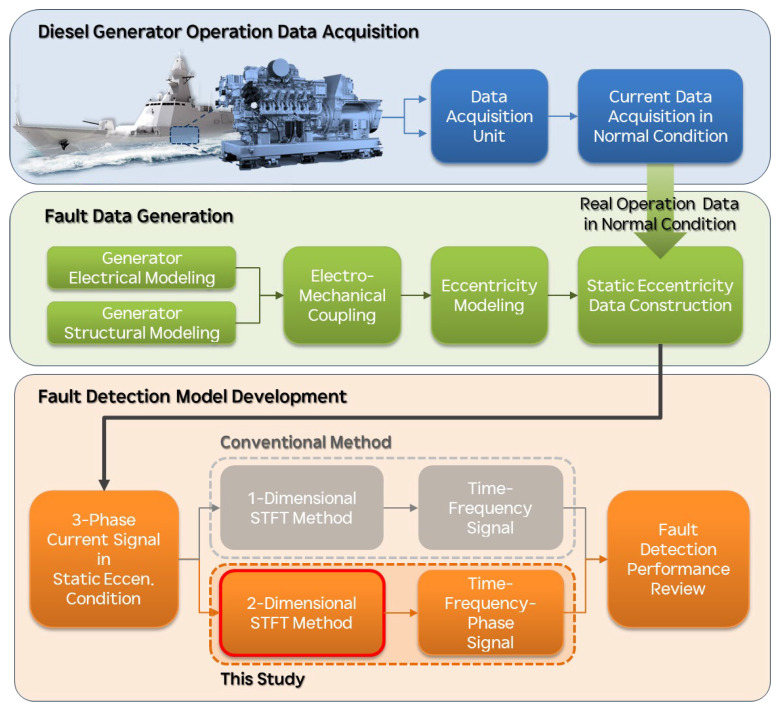
Workflow of the study including data acquisition, fault data generation, and fault detection model development.

Figure 1 depicts the overall workflow of this study, which consists of three main stages: data acquisition, fault data generation, and fault detection model development. In the first stage (data acquisition), real operation current signals are captured from the vessel’s diesel-generator unit through a dedicated data-acquisition (DAQ) unit installed onboard. These long-term measurements under normal conditions provide the reference dataset, denoted as “Current Data Acquisition in Normal Condition.” In the second stage (fault data generation), the normal-condition signals serve as the baseline input for a physics-based simulation chain that integrates generator electrical modeling, structural modeling, and electro-mechanical coupling. Through this coupled framework, static eccentricity is imposed on the generator model to construct “Three-Phase Current Signals in Static Eccentricity Conditions,” enabling systematic synthesis of abnormal data across various eccentricity levels. In the final stage (fault-detection modeling), both the real operation data and the synthesized eccentricity data are analyzed to evaluate diagnostic performance. Conventional approaches typically employ a 1D-STFT to obtain time–frequency representations of each phase independently. In contrast, the present study introduces a two-dimensional short-time Fourier transform (2D-STFT) that constructs a time–frequency–phase representation by simultaneously considering the inter-phase relationships of the three-phase current signals. This formulation enhances the sensitivity to asymmetric fault patterns, allowing for more accurate and robust fault detection performance evaluation in the final review stage.

To compare the fault detection performance of the developed 2D-STFT model with conventional approaches for static eccentricity, it is necessary to obtain fault data under various fault conditions. However, in this study, the current signals were measured from diesel generators installed on operational vessels, where actual fault occurrences are extremely rare due to routine inspections and maintenance conducted by the operating fleet. To overcome this limitation, a physics-based model reflecting the generator’s electrical and mechanical characteristics was developed. This model allows for systematic derivation of current signal variations corresponding to different fault levels, which are then superimposed onto the measured normal-condition signals to synthesize diverse fault scenarios. Static eccentricity, defined as an asymmetric air-gap distribution caused by misalignment between the rotor and stator centers, directly influences both the generator’s electromagnetic forces and current waveforms. By employing a physics-based electro-mechanical coupling model (structure–electric interaction model), the synthesized signals under normal conditions deviated by less than 1% from the rated specifications, demonstrating that the generated fault data closely match the real operating conditions.

### 2.1. Electrical Modeling

The electromechanical model used in this study is based on the multiple coupled circuit (MCC) framework, which accurately captures the interaction between stator windings, rotor excitation, and spatial harmonics. The stator voltage equation is expressed as [12]:

(1) $$V_{s}=R_{s}I_{s}+\frac{\left(d\Psi_{s}\right)}{dt},$$

where $V_{s}$, $I_{s}\;$ and $\Psi_{s}$ represent the stator voltage, current, and flux linkage vectors, respectively. The flux linkage $\Psi_{s}$ is composed of the stator current and the permanent magnet flux, as shown in (2):

(2) $$\Psi_{s}=L_{ss}I_{s}+\Psi_{m},$$

where $L_{ss}$ contains self- and mutual inductances reflecting the stator-slot geometry and winding configuration, and $\Psi_{m}$ represents the permanent-magnet flux (or excitation flux) of the generator. Key assumptions include linear magnetic behavior around the nominal operating point, balanced three-phase supply, and negligible end effects for the frequency band of interest (up to ~2 kHz). Under static eccentricity, the inductance matrix $L_{ss}$ becomes time-varying due to the change in local air-gap length. This modulation introduces additional spatial harmonics that propagate into the current waveforms.

### 2.2. Structural Modeling

The mechanical subsystem models the rotor–bearing–housing assembly using nonlinear bearing stiffness and restoring force characteristics. The bearing reaction forces are expressed as [13]:

(3) $$F_{x}=k(\theta_{i})\delta_{x},F_{y}=k(\theta_{i})\delta_{y},$$

where $k(\theta_{i})$ is the angular position–dependent stiffness of the i-th rolling element, and $\delta_{x}$, $\delta_{y}$ are local deformations in the radial directions. Static eccentricity is introduced by imposing an offset $\left(e_{x},e_{y}\right)$ between the rotor and stator centers, causing non-uniform radial forces. The mechanical equations of motion are:

(4) $$M\ddot{x}+C\dot{x}+K(\theta )x=F_{\mathrm{em}},$$

where $F_{\mathrm{em}}$ is the electromagnetic force computed from the electrical subsystem via the Maxwell stress tensor.

### 2.3. Electro-Mechanical Coupling and Eccentricity Modeling

Static eccentricity is introduced by defining the air-gap length as:

(5) $$g(\theta )=g_{0}(1-ecos(\theta -\theta_{e})),$$

where $g_{0}$ is the nominal air-gap length, e is the eccentricity ratio (1–5% in this study), and $\theta_{e}$ is the angular location of minimum air gap. The air-gap permeance becomes:

(6) $$\Lambda (\theta )=\frac{\mu_{0}}{g(\theta )},$$

leading to spatially varying flux density:

(7) $$B(\theta )\approx \Lambda (\theta )(i_{s}(\theta )+i_{r}(\theta )).$$

The Maxwell stress tensor is then used to compute the electromagnetic forces:

(8) $$F_{r}(\theta ),F_{t}(\theta )\propto \frac{B^{2}(\theta )}{2\mu_{0}}.$$

These forces modulate the inductance matrix and current harmonics. For the studied machine, the resulting harmonic modulation occurs at ~105–110 Hz intervals, with the first prominent overlap with existing machine harmonics occurring at 1020 Hz (17th harmonic)—the key diagnostic signature in this study.

Static eccentricity is introduced in the coupled model by displacing the rotor center with respect to the stator center, which modifies the air-gap permeance distribution and, consequently, the local flux density. The resulting electromagnetic forces are obtained via the Maxwell stress tensor and fed back into the structural model through bearing stiffness coefficients. The electrical model incorporates the actual pole pair number and stator-slot configuration of the target generator, ensuring that the interaction between the eccentric air gap and the spatial harmonics of the stator winding is preserved. Under healthy conditions, the modeled current spectrum reproduces the measured fundamental and design-related harmonic components within approximately 1% magnitude deviation, serving as a baseline validation of the model. When static eccentricity is added, the model predicts harmonic variations occurring at approximately 105–110 Hz intervals, with the first significant interaction appearing at 1020 Hz (17th harmonic of the 60 Hz supply). For the present generator, this 1020 Hz component exhibits the largest and most monotonic increase with eccentricity level, and it is therefore selected as the primary diagnostic indicator. The key parameters incorporated into the coupled electrical–mechanical model include the pole-pair number, stator-slot configuration, nominal air-gap length, and the bearing stiffness coefficients that govern rotor–bearing interaction. These machine-specific parameters ensure that the electromechanical coupling and resulting harmonic modulation—particularly near the 17th harmonic—accurately reflect the physical characteristics of the target generator.

## 3. 2D-STFT-Based Fault Analysis Methodology

This section details a 2D STFT analysis method for detecting static eccentricity using three-phase current data. Conventional 1D STFT treats each phase independently to extract time–frequency information, but it cannot capture inter-phase information or spatial patterns. Static eccentricity arises from an asymmetric air gap between the rotor and stator and induces subtle changes in the phase relationships among the three currents. Such changes are difficult to observe with single-phase time series analysis, underscoring the need for an approach that explicitly considers inter-channel correlations.

Accordingly, we construct a single two-dimensional phase–time signal from the three-phase currents and apply 2D STFT to it, enabling the extraction of features in the time–frequency–phase domain. Compared with conventional methods, this formulation provides a richer representation and, in particular, facilitates quantitative analysis of changes in specific frequency components attributable to faults.

### 3.1. Three-Phase–Time Sequence Construction

The three-phase currents are denoted as $I_{u}(t)$, $I_{v}(t)$ and $I_{w}(t)$. Arranging these along the time axis in matrix form yields:

(9) $$I\left(t\right)=\left[\begin{array}{cc} \begin{array}{cc} I_{u}(t_{1}) & I_{u}(t_{2})\\ I_{v}(t_{1}) & I_{v}(t_{2})\\ I_{w}(t_{1}) & I_{w}(t_{2}) \end{array} & \begin{array}{cc} \ldots & I_{u}(t_{N})\\ \ldots & I_{v}(t_{N})\\ \ldots & I_{w}(t_{N}) \end{array} \end{array}\right]$$

This two-dimensional representation incorporates both the time axis and the phase axis, preserving inter-phase relationships at each time instant. Beyond simple data arrangement, this structure provides a foundation for quantitatively analyzing phase interactions under fault conditions. Static eccentricity does not affect only a single phase; rather, it induces subtle variations across the entire current distribution. Therefore, extending the analysis to a two-dimensional sequence is considered a suitable approach for accurate fault diagnosis.

### 3.2. Definition of 2D STFT

The 2D STFT extracts localized time–frequency information from a two-dimensional signal and is defined as:

(10) $${STFT}_{2D}\left(x,y,f_{x},f_{y}\right)=\iint I(x^{\prime },\;y^{\prime })\cdot w(x^{\prime }-x,y^{\prime }-y)\cdot e^{-j2\pi \left(f_{x}x^{\prime }+f_{y}y^{\prime }\right)}dx^{\prime }dy^{\prime }$$

- $I\left(x^{\prime },\;y^{\prime }\right)$: input 2D sequence

- $w\left(x,\;y\right)$: 2D window function

- $f_{x},f_{y}$: frequency components along the time and phase axes

Unlike 1D STFT, which extracts frequency content only along the time axis, 2D STFT analyzes both the time and phase dimensions, providing a richer representation of the signal. In particular, the phase axis frequency components are highly effective for capturing inter-phase interactions and spatial patterns associated with faults such as static eccentricity [14,15].

### 3.3. Parameter Configuration for STFT

The performance of 2D STFT depends on various parameter choices such as window size, overlap ratio, and FFT length [15]. Each parameter must be carefully selected to balance the tradeoff between time and frequency resolution. In this study, the parameters were configured as summarized in Table 1 to ensure effective detection of static eccentricity faults using real diesel generator current data.

The selection of STFT parameters was based on the spectral characteristics of the target generator. A 1 s window length was chosen to ensure sufficient frequency resolution, as it contains 60 cycles of the 60 Hz fundamental component and therefore provides stable estimation of harmonic magnitudes. The 10% overlap offers a practical balance between temporal continuity and computational cost while avoiding excessive redundancy given the quasi-stationary operating condition of the generator. A Hann window was used because of its favorable sidelobe suppression properties, which reduce spectral leakage and improve the ability to distinguish subtle eccentricity-induced variations near the 1020 Hz harmonic. These parameter choices were verified to provide clear separation of harmonic components without introducing smoothing artifacts that could obscure early-stage fault signatures.

## 4. Experimental Setup and Analysis Results

### 4.1. Data Acquisition and Measurement System

**Figure 2:**
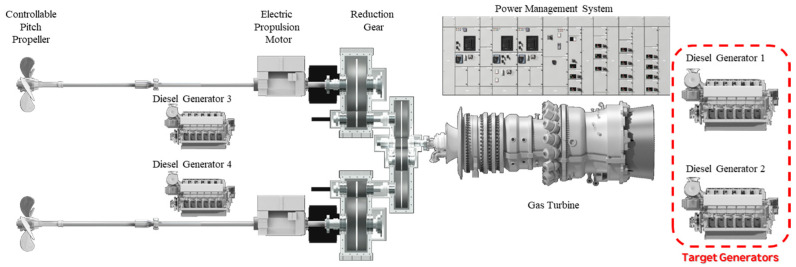
Configuration of the CODLOG propulsion system (target system for current data acquisition).

Figure 2 illustrates the overall propulsion system configuration of the vessel, which adopts a CODLOG (Combined Diesel-Electric or Gas) arrangement integrating diesel-electric propulsion and a gas-turbine drive. In this configuration, multiple diesel generators supply electric power to the propulsion motors and auxiliary systems during normal cruising and low-to-medium-speed operations, ensuring high efficiency and operational flexibility. At higher speeds, propulsion switches from the electric drive to a gas turbine that is mechanically coupled through a reduction gearbox, providing the necessary thrust for rapid acceleration and high-speed operation. This arrangement allows the propulsion system to selectively utilize either diesel-electric or gas-turbine modes, achieving both redundancy and fuel efficiency without the need for simultaneous operation.

Among the components of the CODLOG system, the diesel generator plays a pivotal role as the principal power source under most operating conditions. It not only provides stable electrical energy to the propulsion motors but also serves as a critical element influencing the overall reliability and performance of the propulsion system. Therefore, the present study focuses on the diesel-generator unit to analyze its electrical characteristics, monitoring data, and potential fault behaviors under real operating conditions.

In the CODLOG propulsion system of the target vessel, four diesel-generator units are installed. The number of generators in operation varies depending on the power demand under different mission conditions. Specifically, Generator #1 and Generator #2 are located in the fore section of the vessel, while Generator #3 and Generator #4 are positioned in the aft section. In this study, three-phase current measurements were obtained from Generator #1 and Generator #2 installed in the fore section.

**Figure 3:**
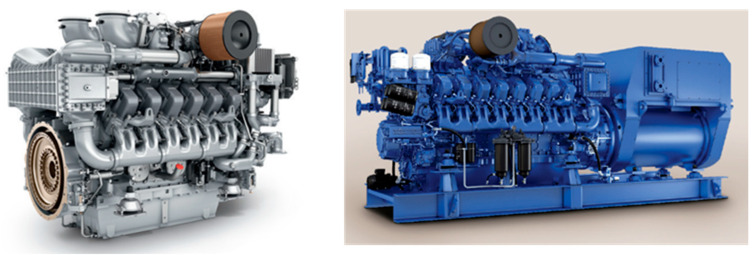
Target diesel engine, MTU 12V 4000 (**left**), and a typical diesel generator configuration with alternator coupling (**right**) [16].

Figure 3 presents the external configuration of the target diesel generator, and Table 2 summarizes its principal specifications. The target generator is an MTU 12V 4000 series unit, a high-performance 12-cylinder V-type diesel engine widely used in marine propulsion and onboard power-generation systems. It is designed to provide high output power with excellent fuel efficiency and operational stability, making it suitable for continuous service in demanding marine environments. The generator operates at a rated output of 1650 kW, 450 V, and 60 Hz, supplying primary electrical power for both propulsion and auxiliary equipment on board. The left image in Figure 3 shows the actual MTU 12V 4000 diesel engine (MTU Friedrichshafen GmbH, Friedrichshafen, Germany) used in this study, while the right image illustrates a typical generator configuration in which the diesel engine is coupled with an alternator for power generation.

**Table 2:** Specifications of Target Diesel Generator used for current signal measurement and analysis.

Parameter	Value
Generator/Engine Model	MTU 12V 4000 series diesel generator (based on 12V 4000 G14F/DS1650)
Rated Output	1650 kW at 60 Hz (three-phase)
Voltage	450 V (line-to-line)
Frequency	60 Hz
Speed	1800 rpm
Cylinder Configuration	12 V configuration, 4-stroke, turbocharged diesel engine
Bore × Stroke	170 mm × 210 mm
Displacement	57.2 L
Specific fuel consumption	≈200 g/kWh (at 100% load, ISO 3046-1 [17])
Dimensions (L×W×h)	4059 mm × 1810 mm × 2330 mm (open power unit)
Dry weight	10,654 kg (open power unit)

The experimental subject of this study is an actual marine diesel generator. The rated values of the generator are 1650 kW, 450 V, at 60 Hz, and three-phase current data were collected under normal operating conditions. Current signals were measured using non-contact current transducers (CTs) installed at the output terminals and recorded with a DAQ (Data Acquisition) system at a sampling frequency of 10.417 kHz, thereby obtaining three-phase waveforms representative of normal operation.

**Figure 4:**
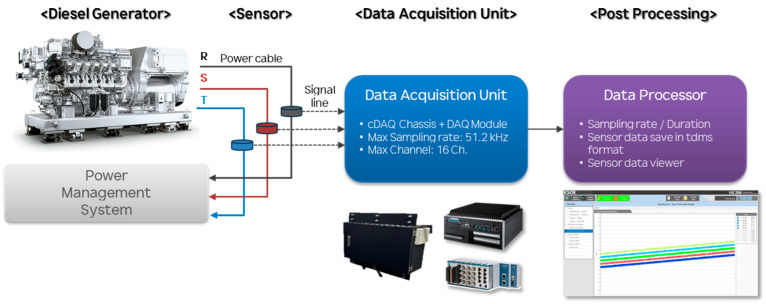
Schematic diagram of the current data acquisition system for the target diesel generator.

Figure 4 illustrates the overall framework of the data flow employed in this study, which comprises four sequential stages: diesel generator operation, signal measurement through three-phase current sensors, data acquisition, and post-processing. In the first stage, three-phase output currents generated by the diesel generator are captured using non-contact CTs mounted on the generator output terminals. Table 3 summarizes the detailed specifications of the CTs used in the measurement setup. Unlike conventional CTs, which provide only time-averaged RMS values, the sensors employed in this study are designed to deliver high-resolution raw current data suitable for frequency-domain analysis, thereby enabling precise fault-diagnosis applications. Each phase (R, S, and T) is measured independently to preserve the temporal and phase-angle relationships among the three signals.

In the second stage, the analog current signals are transmitted to the DAQ unit, which consists of a Compact DAQ (cDAQ) chassis integrated with modular DAQ boards. This configuration supports a maximum sampling rate of 50 kHz per channel and accommodates up to 32 input channels, allowing for simultaneous multi-channel signal recording with high fidelity. The DAQ unit performs analog-to-digital conversion and stores the digitized signals for subsequent analysis. To ensure stable data collection during continuous long-term monitoring, system synchronization and sampling-rate control were implemented through a dedicated signal-conditioning interface.

In the final stage, the recorded current data are processed through a data-processing environment, referred to as the “Data Processor” in Figure 4. Within this module, the sampling rate and acquisition duration are configured according to the experimental requirements, and the acquired sensor data are saved in the Technical Data Management Streaming (TDMS) format to facilitate efficient storage and retrieval of large time-series datasets. A dedicated sensor-data viewer is incorporated into the processor to enable real-time visualization, verification, and preliminary quality assessment of the acquired waveforms prior to advanced signal analysis. This structured data-flow configuration ensures reliable high-frequency signal acquisition from the generator and provides a robust foundation for the subsequent modeling and fault-detection processes.

**Figure 5:**
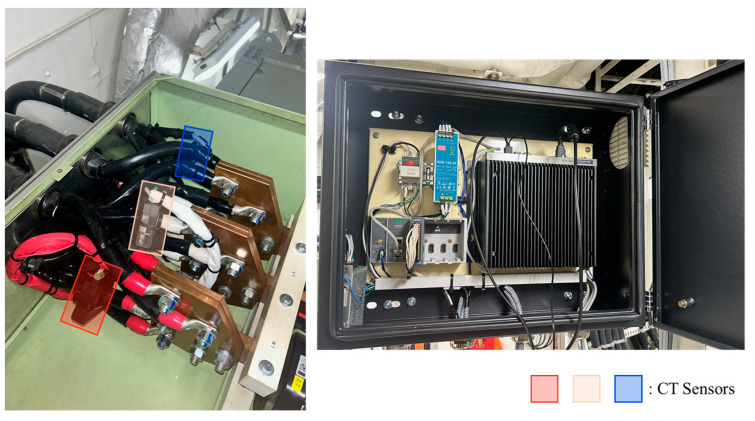
Experimental setup of the current measurement system for the target diesel generator: (**Left**) installation of R, S and T current transducers inside the generator enclosure; (**Right**) DAQ module used for current signal recording.

Figure 5 represents the experimental setup of the current measurement system, showing the non-contact CTs mounted on the generator output terminals and their connection to the DAQ unit. In this configuration, individual sensors are installed on each phase—R, S and T—thereby allowing for simultaneous acquisition of all three-line currents. Each phase’s analog output is then routed into the DAQ module, ensuring that the temporal relationship and phase angle between R, S and T currents are preserved for subsequent signal processing and fault-detection analysis.

**Figure 6:**
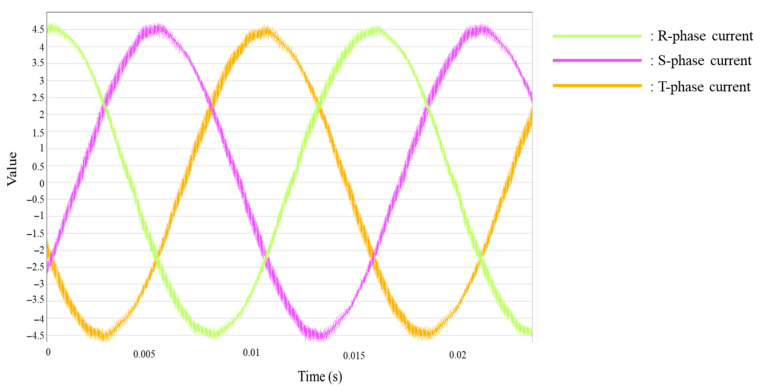
Example of measured three-phase current time-series under normal operating conditions.

Current data were collected at a sampling rate of 10.417 kHz, and long-term three-phase current measurements obtained over three months were used as the reference for normal operating conditions. A representative time-series waveform of the measured current signals is presented in Figure 6, illustrating the stable and periodic behavior of the generator under steady-state operation. It should be noted that the generator output consists of six parallel conductors per phase, while the current was measured from a single conductor. Therefore, the actual line current corresponds to approximately six times the measured value. Fault scenario data were generated by taking the measured normal condition signals, imposing static eccentricity levels of 1–5%, and then applying these conditions to the physics-based model for signal synthesis.

### 4.2. Construction of Fault Data Using Real Operation Measurements

**Figure 7:**
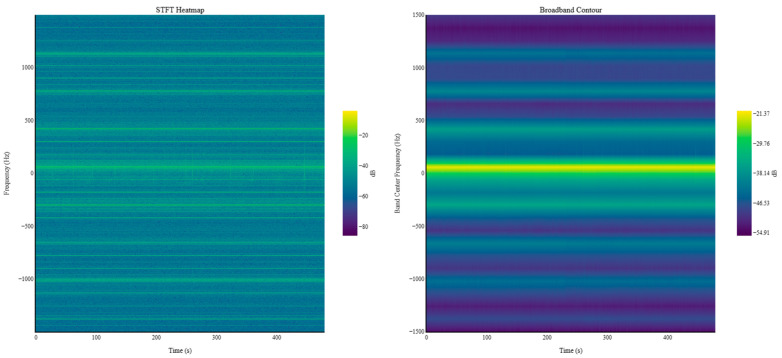
(**Left**) STFT spectrogram of the measured current signal and (**Right**) its broadband energy contour.

Figure 7 (left) shows the STFT spectrogram of the current signal measured from the target diesel generator under steady normal operating conditions. Because the generator was operating in a quasi-steady state without significant variations in load or operating conditions, the resulting time–frequency distribution remains stable over time, exhibiting no abrupt changes in spectral characteristics. The right panel of Figure 7 presents the broadband energy contour obtained by dividing the frequency axis into 30 Hz intervals and computing the power spectral density within each band. This representation allows for clear identification of the frequency regions where the signal energy is concentrated. The dominant energy appears at 60 Hz, corresponding to the rotational frequency of the generator, with additional prominent components at the 7th harmonic (420 Hz) and the 13th harmonic (780 Hz). Such harmonic patterns are commonly determined by key alternator design parameters—such as the number of stator slots, winding configuration, and coil span—and the pronounced energy concentrations observed at the 7th and 13th harmonics are attributed to the inherent electromagnetic characteristics of the alternator installed on the target diesel generator.

**Figure 8:**
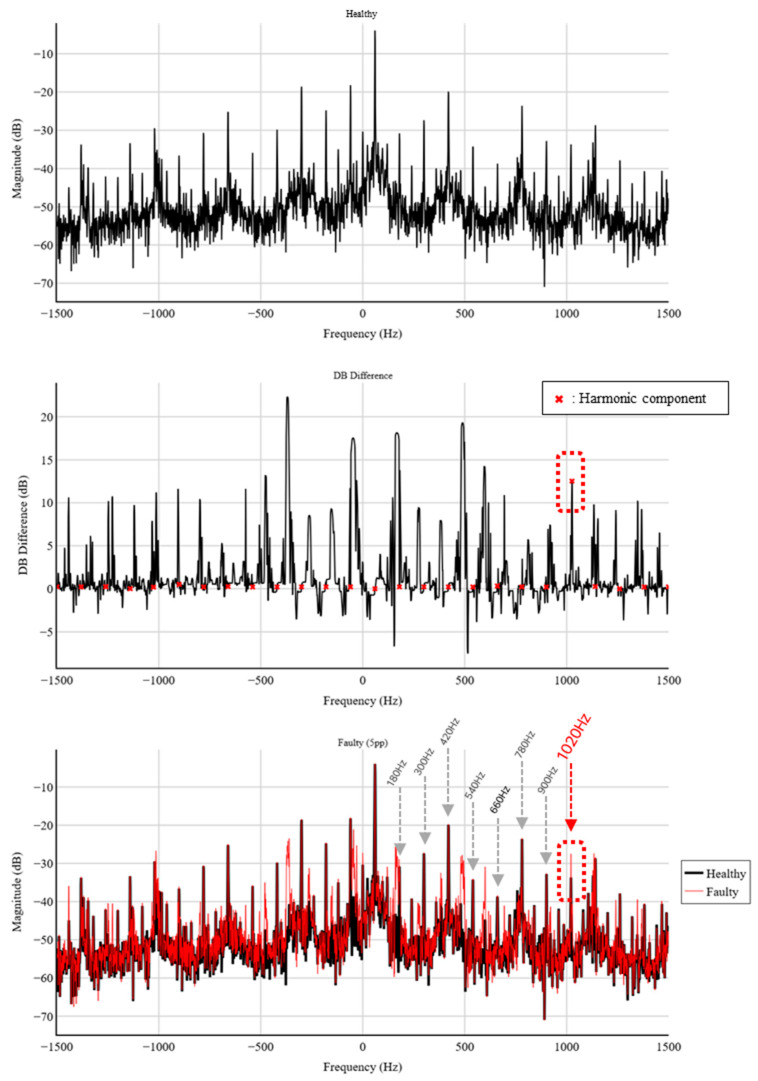
Static-eccentricity fault data construction using the physics-based generator model (**Top**) Normal-condition data (**Middle**) dB variation for each frequency component (**Bottom**) Final fault-simulated data.

Figure 8 presents the process of constructing static-eccentricity fault data by applying the physics-based model to the healthy current measurements obtained from the target diesel generator. The top panel shows the 2D STFT representation of the measured current signal under steady operating conditions. Although the spectral characteristics appear similar to those of the previously shown STFT spectrogram, the 2D STFT considers inter-phase variations among the three-phase currents; as a result, the frequency distribution extends from negative to positive frequencies and exhibits a left–right asymmetric pattern. Consistent with earlier observations, the dominant peak occurs at the 60 Hz rotational frequency, followed by additional peaks at its harmonic components. In particular, due to the alternator design, pronounced peaks are observed at the 6th and 12th harmonic orders.

The middle panel in Figure 8 displays the simulated effect of static eccentricity obtained from the physics-based generator model. This plot shows the dB variation for each frequency component when a static eccentricity fault occurs. Static eccentricity causes periodic fluctuations in the air gap between the stator and rotor, and the resulting electromagnetic behavior manifests as characteristic changes across specific frequency components. Among these components, those aligned with the rotational frequency and its harmonics exhibit meaningful deviations, whereas non-harmonic components—which are negligible in the healthy signal—do not produce significant differences even when fault-induced components are added. Examination of the simulated variation profile indicates that although multiple peaks appear across the spectrum, the first harmonic order that coincides with the generator’s harmonic structure is located at 1020 Hz, corresponding to the 17th harmonic. Therefore, the 1020 Hz component demonstrates the most distinguishable difference between healthy and faulty conditions and is identified as the key diagnostic frequency for static eccentricity.

The bottom panel of Figure 8 illustrates the frequency characteristics of the final static-eccentricity fault–simulated data, obtained by superimposing the fault-induced spectral variations onto the healthy measurement. As shown, most harmonic components exhibit minimal change before and after fault injection, whereas the 1020 Hz component demonstrates a pronounced increase, consistent with the model-predicted fault effect. In this study, static eccentricity levels from 1% to 5% were applied to generate multiple fault-simulation cases, which were subsequently used for validating the proposed fault-detection model. It is noted that the synthesized fault data used in this study represent model-derived approximations of static eccentricity rather than real fault measurements. This hybrid approach enables systematic control of fault severity at very low eccentricity levels but does not substitute field measurements from actual eccentricity events. Accordingly, the selected eccentricity range of 1–5% represents a practically relevant and literature-supported regime for assessing early-stage detection performance [10,11].

### 4.3. One-Dimensional STFT Analysis Results (Conventional Method)

**Figure 9:**
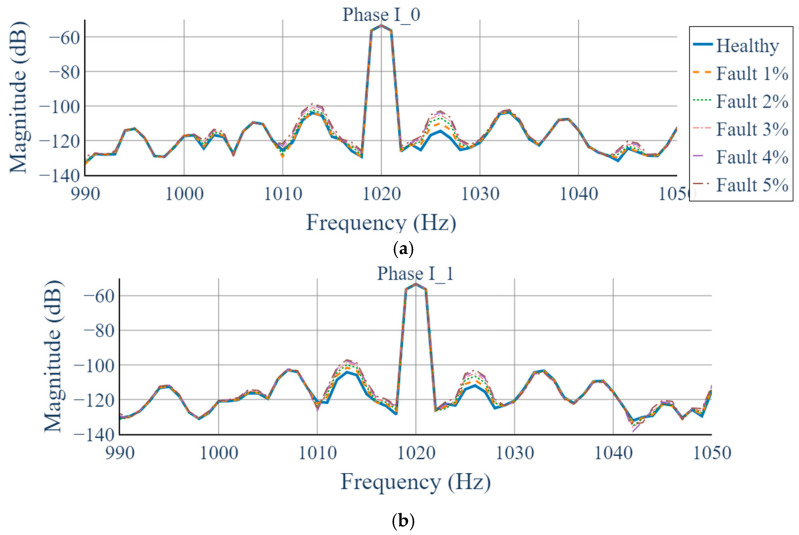

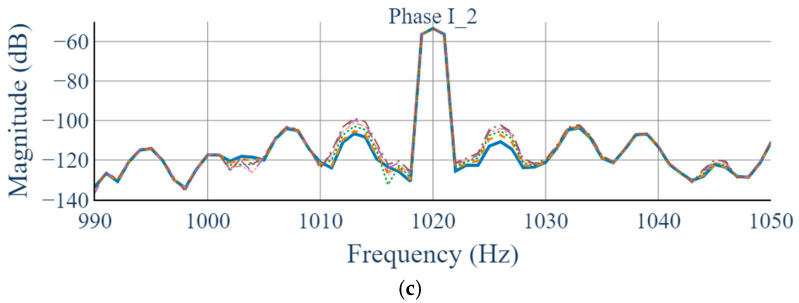
Comparison of 1D STFT results of phase currents under different degrees of static eccentricity: (**a**) Phase-R (**b**) Phase-S (**c**) Phase-T.

The conventional 1D STFT processes each phase current independently to extract time–frequency information. However, the analysis revealed no clear spectral distinction between normal and static eccentricity conditions; the energy distributions of the two datasets appeared nearly identical, making fault identification difficult. As shown in Figure 9, the frequency region around 1020 Hz—where fault-induced variations are expected—exhibits almost no visible difference between healthy and faulty signals. This limitation arises because 1D STFT analyzes each phase separately and therefore cannot capture inter-phase relationships. Any fault-related variations that appear as differences between phases at the same temporal frequency remain hidden within single-phase spectrograms. Consequently, the 1D STFT approach shows limited sensitivity to static eccentricity faults.

### 4.4. Two-Dimensional STFT Analysis Results (Proposed Method)

The limitations identified in the 1D STFT results highlight the need for an analysis method that can incorporate inter-phase relationships. To address this, the proposed 2D STFT introduces the phase axis into the time–frequency representation, enabling fault-induced asymmetries to be more clearly exposed.

**Figure 10:**
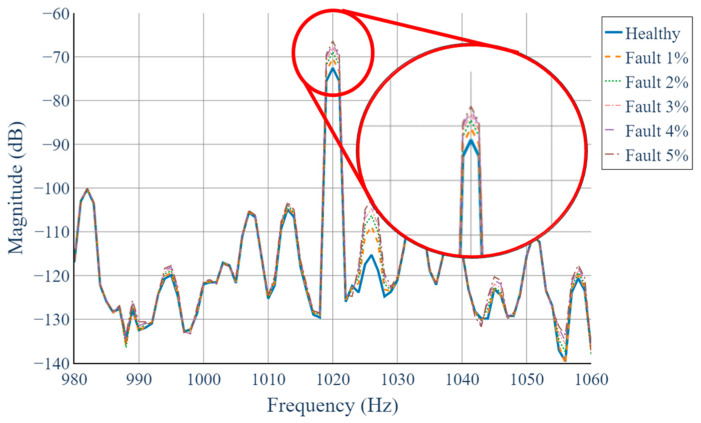
Comparison of 2D STFT results in the time–phase domain under different degrees of static eccentricity.

Figure 10 presents the 2D STFT results in the time–phase domain under different levels of static eccentricity. As the eccentricity percentage increases from 1% to 5%, the harmonic component near 1020 Hz becomes progressively more pronounced. This visual trend demonstrates that the 2D STFT effectively captures the fault-induced modulation that is not discernible in conventional single-phase analyses.

The quantitative variation observed in these harmonic components is summarized in Table 4. The 1020 Hz component corresponds to the 17th harmonic of the generator’s 60 Hz supply frequency, reflecting the spatial periodicity of the air-gap asymmetry caused by static eccentricity. For the tested generator, this 17th harmonic shows a monotonic increase in magnitude with fault severity, indicating its suitability as a diagnostic indicator. It should be noted that the dominant harmonic order may vary depending on alternator design characteristics.

**Figure 11:**
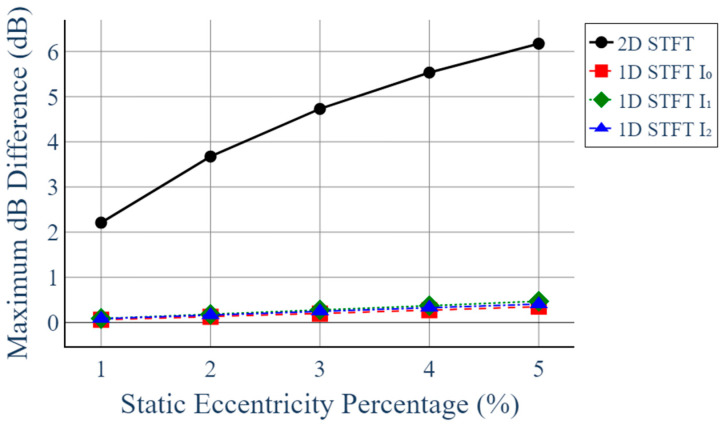
Comparison of Maximum dB Difference with Respect to Static Eccentricity in 1D and 2D STFT Analyses.

Figure 11 further compares the maximum dB differences obtained from 1D and 2D STFT analyses. While the three single-phase 1D STFT results (I_0_, I_1_, I_2_) show only minimal changes across all fault levels—remaining below approximately 0.5 dB—the 2D STFT exhibits a steadily increasing response exceeding 6 dB at 5% eccentricity. This confirms the superior sensitivity of the proposed 2D STFT in detecting static eccentricity faults.

For practical implementation, we define a normalized indicator $\Delta H_{1020}$ as the difference in dB between the 2D STFT magnitude at 1020 Hz and its healthy baseline value at the same operating point. In the present study, $\Delta H_{1020}$ increases from approximately 0 dB (0% eccentricity) to more than 6 dB at 5% eccentricity, while the corresponding variation in the single-phase 1D STFT results remains below about 0.5 dB. A simple detection rule can therefore be formulated by setting a threshold on $\Delta H_{1020}$ (e.g., 3 dB) to flag a potential static eccentricity fault. In practice, this threshold would be tuned for each generator and operating condition during commissioning. A detailed robustness and threshold-optimization study under different load and noise conditions is beyond the scope of this work and will be addressed in future research.

From a practical implementation perspective, the proposed method requires only the three-phase currents that are already monitored in most marine power-generation systems, enabling a fully non-intrusive diagnostic workflow. Although the present study focused on the load condition most frequently used by the target generator, preliminary tests on additional operating points indicated that the 1020 Hz indicator exhibits similar monotonic behavior, with differences mainly in absolute magnitude. In real shipboard applications, a healthy baseline and operating-condition–specific thresholds would therefore be established during commissioning for each major load region. For vessels without historical data, the distribution of Δ*H*_1020_ across a fleet of similar generators could provide a population-based reference interval. A comprehensive assessment of robustness under varying load profiles, noise environments, and supply disturbances remains an important direction for future work.

## 5. Conclusions

This paper presented a novel diagnostic methodology for detecting static eccentricity faults in marine diesel generators using a 2D STFT applied to experimentally measured three-phase current signals. The novelty of this study lies in extending conventional one-dimensional time–frequency analysis into a multidimensional time–phase–frequency representation, enabling simultaneous interpretation of temporal, spectral, and inter-phase features within a unified analytical framework. Previous approaches have generally analyzed each phase independently, overlooking spatial and inter-phase dependencies. In contrast, incorporating the phase dimension into the transform significantly enhances diagnostic sensitivity to electro-mechanical coupling phenomena such as eccentricity.

A comprehensive diagnostic process was developed, encompassing experimental data acquisition, model-based fault synthesis, and frequency–domain feature extraction. Three-phase current signals were experimentally obtained from an MTU 12V 4000 marine diesel generator (1650 kW, 450 V, 60 Hz) under nominally healthy operating conditions. Static eccentricity signals were then synthesized by combining the results of a physics-based electro-mechanical coupling model (representing 1–5% eccentricity) with the measured healthy current waveforms, thereby generating realistic fault scenarios that preserve the electrical and dynamic characteristics of the actual machine. The three-phase currents were organized into a two-dimensional phase–time matrix, to which 2D STFT was applied for joint time–frequency–phase analysis and compared with conventional 1D STFT results.

The 2D STFT analysis demonstrated clear diagnostic superiority. While the 1D STFT exhibited minimal spectral distinction between normal and faulted conditions, the 2D STFT revealed a pronounced energy component near 1020 Hz, corresponding to the 17th harmonic of the 60 Hz supply frequency, whose amplitude increased systematically with the eccentricity ratio. This harmonic component served as a stable and quantifiable indicator of static eccentricity, allowing for reliable detection even at the 5% eccentricity level. Quantitatively, the 2D STFT spectra showed measurable energy differences of several decibels between healthy and faulted states, whereas the 1D analysis remained largely invariant. By leveraging inter-phase correlation structures among the three-phase currents, the proposed 2D STFT framework effectively captures spatial–temporal interactions that are unobservable in single-channel analyses. Because the method relies solely on existing current measurements, it enables fully non-contact fault diagnosis without requiring any additional sensors or intrusive instrumentation. This multi-dimensional representation enhances fault sensitivity, noise robustness, and diagnostic precision, establishing 2D STFT-based current-signal analysis as a non-contact, data-driven, and computationally efficient tool for early detection of rotor–stator air-gap asymmetry in marine diesel generators.

Despite these promising results, the present study should be regarded as an early-stage proof of concept due to several inherent limitations in the validation process. The evaluation relied on hybrid datasets created by superimposing model-derived eccentricity effects onto healthy measurements from a single generator, and the analysis was conducted under quasi-steady operating conditions without access to real in-service eccentricity events. As a result, the diagnostic sensitivity demonstrated here cannot yet be generalized across different machine types, alternator designs, or varying load and noise environments. These factors collectively highlight the need for broader empirical verification before the method can be applied in operational marine settings.

Nonetheless, the hybrid-modeling results confirm the technical feasibility and diagnostic potential of the 2D STFT framework for early-stage eccentricity detection. Future work will extend validation to in-service measurements from multiple vessels; broaden applicability to dynamic eccentricity, bearing degradation, and insulation deterioration; and incorporate adaptive thresholding and machine-learning-based decision frameworks. By enriching both empirical validation and analytic automation, the proposed method can evolve into a deployable component of AI-driven condition-based monitoring and predictive maintenance in next-generation marine propulsion and power-generation systems.

## Figures and Tables

**Table 1:** STFT parameter settings for current signal analysis.

Parmeter	Value
Window Size	10,417 × 3 phase
Overlap	10%
Window Function	Hann

**Table 3:** Specification of CT Sensor.

Parameter	Value
Model	AT 300
Current Measurement Range	300 A (DC or AC peak)
Output Sensitivity	10 mV/A
Resolution	±1 mA
Accuracy	±1% of reading ± 5 mA
Bandwidth	DC to 100 kHz (−3 dB)
Max Conductor Diameter	∅ 25 mm
Operating Temperature	−20 °C to +65 °C
Ingress Protection	IP40 (jaw closed)
Supply Voltage	±15 V external ± 10%
Dimensions (H×W×D)	100 mm × 65 mm × 25 mm

**Table 4:** STFT Magnitude at 1020 Hz.

Static Eccentricity	Magnitude (dB)
0%	−72.598
1%	−70.35
2%	−68.893
3%	−67.839
4%	−67.035
5%	−66.398

## Data Availability

Restrictions apply to the availability of the datasets used in this study. The data involve operational and experimental information from defense-related systems and are subject to corporate confidentiality and national security regulations. Therefore, the datasets cannot be made publicly available. Requests to access the data should be directed to the corresponding author and may be considered on a restricted basis.
